# Radiographically Occult Symptomatic Lunotriquetral Coalition in a Child

**DOI:** 10.7759/cureus.5364

**Published:** 2019-08-11

**Authors:** Ravi V Patel, Monica S Epelman, Arthur B Meyers

**Affiliations:** 1 Internal Medicine, University of Central Florida College of Medicine, Orlando, USA; 2 Radiology, Nemours Children's Hospital/University of Central Florida College of Medicine, Orlando, USA

**Keywords:** carpal coalition, lunotriquetral coalition, pediatric

## Abstract

Carpal coalitions are congenital segmentation anomalies with an abnormal union of one or more carpal bones. They can be broadly classified as osseous or non-osseous and as partial or complete. Lunotriquetral coalitions are the most common type of carpal coalition and are typically asymptomatic and detected incidentally. However, there are several case reports and small case series reporting symptomatic non-osseous lunotriquetral coalitions, which show findings on radiographs. This is a report of a case of a child with a symptomatic non-osseous lunotriquetral coalition which was undetected on initial radiographs but diagnosed on a subsequent wrist magnetic resonance imaging (MRI).

## Introduction

Carpal coalitions are congenital anomalies with an abnormal union of one or more carpal bones. Lunotriquetral coalitions are the most common type, accounting for approximately 90% of all carpal coalitions [1]. These coalitions are the result of failure of segmentation of the cartilage precursor of the carpal bones [1-2]. Carpal coalitions can be broadly classified as osseous or non-osseous and are also described as partial or complete [1-2]. Patients with complete osseous union at a carpal coalition are asymptomatic and this is typically found incidentally at imaging for other causes of pain or after a trauma [2]. However, there are several case reports and small case series of symptomatic non-osseous carpal coalitions [2-7]. We present a case of a 13-year-old boy with a symptomatic non-osseous lunotriquetral coalition which was undetected on initial radiographs but diagnosed on a subsequent wrist magnetic resonance imaging (MRI).

## Case presentation

**Figure 1:**
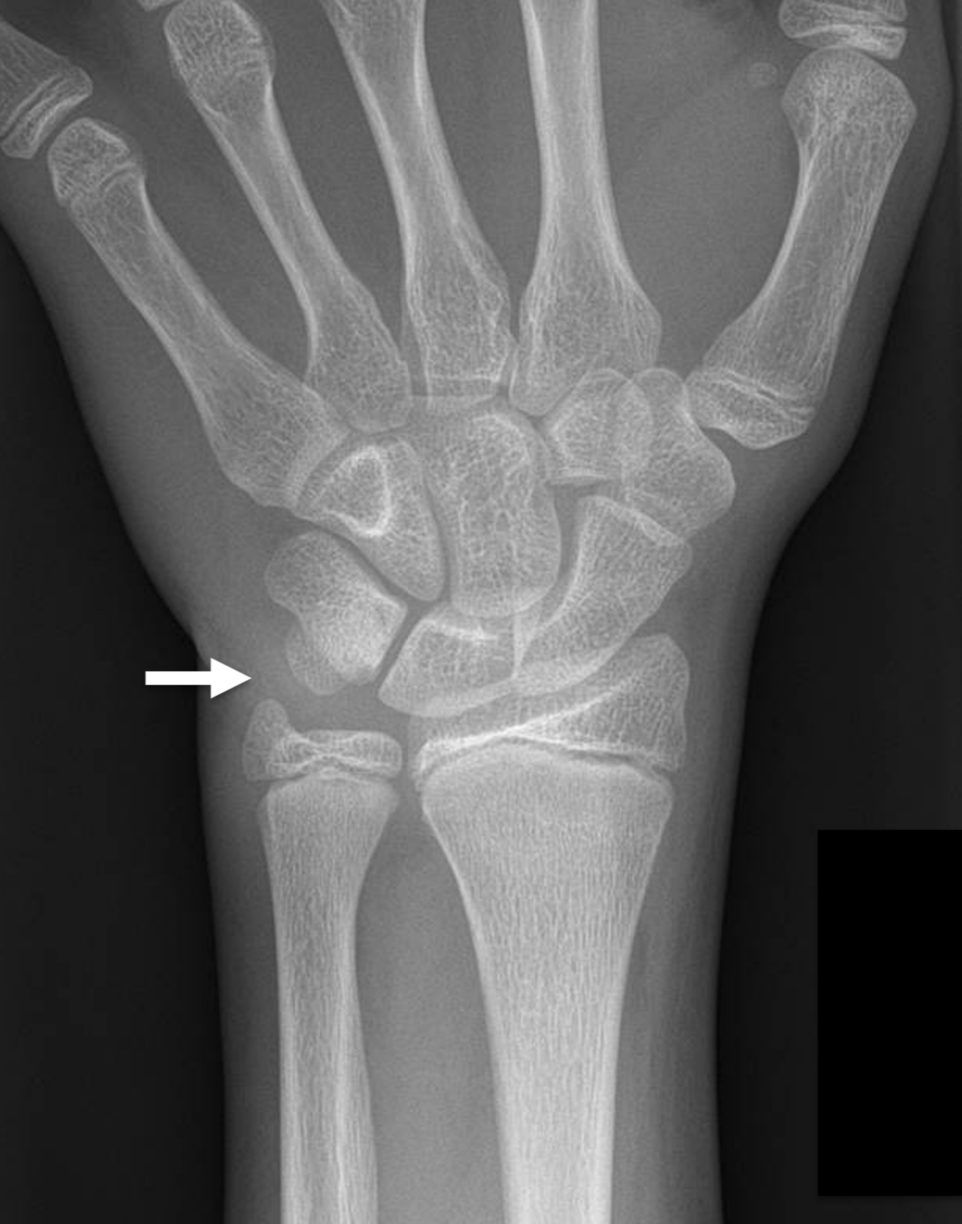
13-year-old boy with chronic ulnar sided left wrist pain This initial frontal radiograph of the left wrist was interpreted as normal. The arrow indicates the area of the patient's reported pain.

A 13-year-old boy presented with left wrist pain when actively using his wrist. He had injured this wrist three months prior when he collided with another player in a soccer game. However, upon further inquiry, it was discovered that he had been experiencing ulnar-sided wrist pain with repetitive use and limited range of motion of the left wrist prior to that injury. The patient had a three-view radiographic series of his left wrist at this time, which showed no definite abnormality (Figure 1). He was placed in a wrist splint and instructed to limit physical activity involving the left wrist. His left wrist pain persisted.

**Figure 2:**
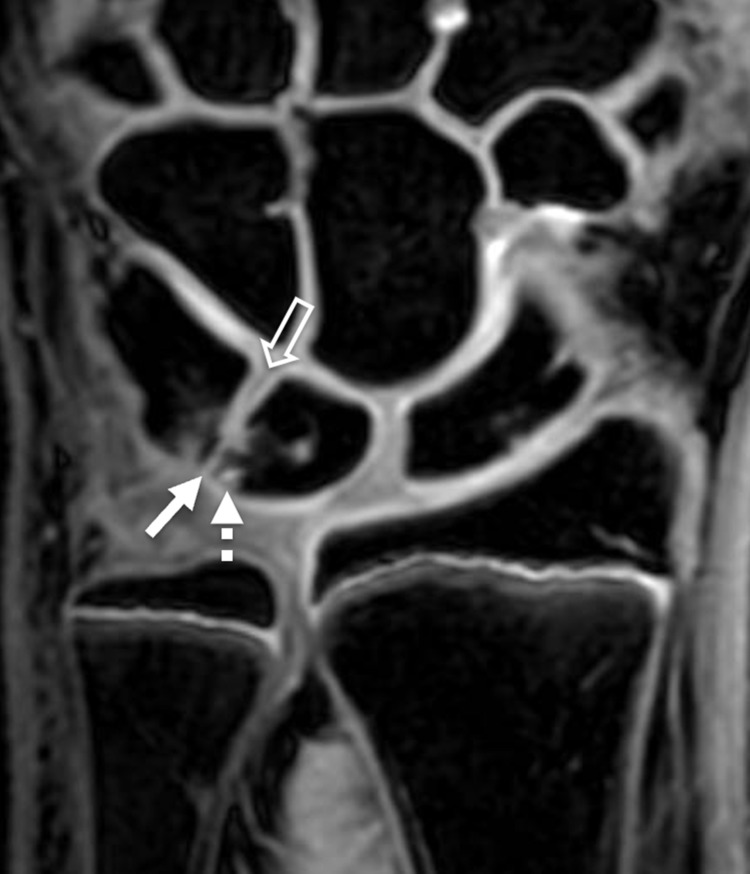
Coronal gradient echo magnetic resonance (MR) image of the left wrist in the same child The MR image shows a partial cartilaginous coalition of the proximal aspect of the lunotriquetral joint (solid arrow). Associated subchondral cystic change is also present (dashed arrow). Notice that the distal portion of the joint appears normally formed (open arrow), indicating that this is a partial coalition.

**Figure 3:**
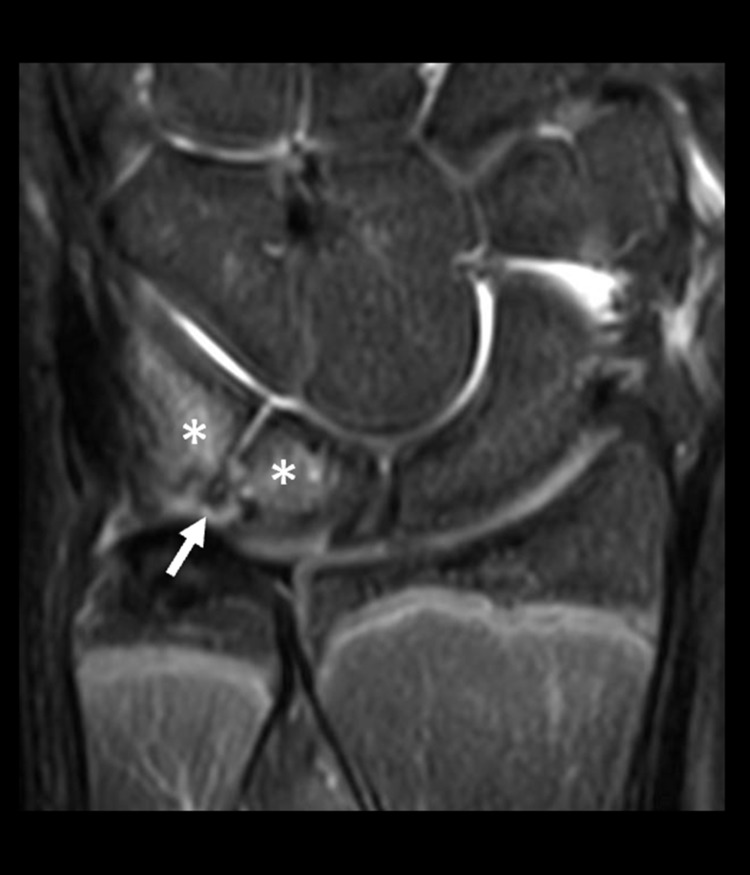
Coronal T2-weighted fat-suppressed magnetic resonance (MR) image from the same MR scan as the prior figure There is edema-like signal (asterisks) within the lunate and triquetrum adjacent to the coalition (solid arrow), consistent with stress changes.

A left wrist MRI was requested and the boy’s left wrist was imaged on a 3T magnetic resonance (MR) scanner. The standard wrist protocol at our institution was performed, which includes the following sequences: coronal T1-weighted, coronal gradient echo, coronal T2-weighted FS, axial T2-weighted FS, sagittal 3D proton density and sagittal T2-weighted FS sequences. The MR images showed an incomplete cartilaginous lunotriquetral coalition with associated irregularity of the margins of the lunate and triquetrum and subchondral cystic changes adjacent to the synchondrosis (Figure 2). Additionally, an edema-like signal was also present in the lunate and triquetrum adjacent to the synchondrosis (Figure 3). This child and his parents did not elect to have surgical partial arthrodesis and he was subsequently lost to follow-up.

## Discussion

Carpal coalitions are congenital anomalies characterized by union of the carpal bones. It is estimated that approximately 0.1% of the general population has some form of carpal coalition, with lunotriquetral coalitions being the most common type [1]. However, various reports have shown wide variation in the prevalence of coalitions in different geographic regions; for instance, the prevalence of lunotriquetral coalitions in persons of West African descent has been reported to be as high as 9.5% [1]. Most carpal coalitions are asymptomatic and are found incidentally while imaging the wrist, hand or forearm for another reason. 

Carpal coalitions occur secondary to a failure of segmentation of the common cartilage precursor of the carpal bones [1-3]. Normally, apoptosis of certain cells within the cartilaginous carpal precursor of the lunate and triquetrum creates a cleft between the lunate and triquetrum by the tenth week of intrauterine life [7]. It is thought that the degree to which this process fails to occur leads to the spectrum of findings seen with lunotriquetral coalitions. Complete failure of apoptosis results in a complete osseous coalition with total absence of the joint and various degrees of partial osseous or fibrocartilaginous coalitions occur when this process is limited or aborted [7]. Resnik et al. injected the mid-carpal row in a patient with a partial lunotriquetral coalition, which confirmed that a small distal portion of the lunotriquetral joint had formed in that patient [4]. At times patients with incomplete coalitions are referred to as having partial or incomplete "fusions". However, this terminology should be avoided because it is inaccurate given that the underlying etiology is failure of segmentation not an acquired fusion of previously distinct bones [3-4]. Acquired fusion of carpal bones may occur secondary to other pathologies, such as the carpal bone ankylosis that can be seen in juvenile idiopathic arthritis, and therefore should be distinguished from carpal coalitions [1].

Most carpal coalitions are found in isolation, but they may be associated with syndromes, skeletal dysplasias and other disorders (e.g., Turner syndrome, Holt-Oram syndrome and Ellis-van Creveld/chondroectodermal dysplasia) [1,8]. When found in isolation, carpal coalitions typically involve two carpal bones in the same carpal row [1]. However, when found with a syndromic association the fusion may be more complex, involving more than two bones and bridging across carpal rows [1]. 

**Figure 4:**
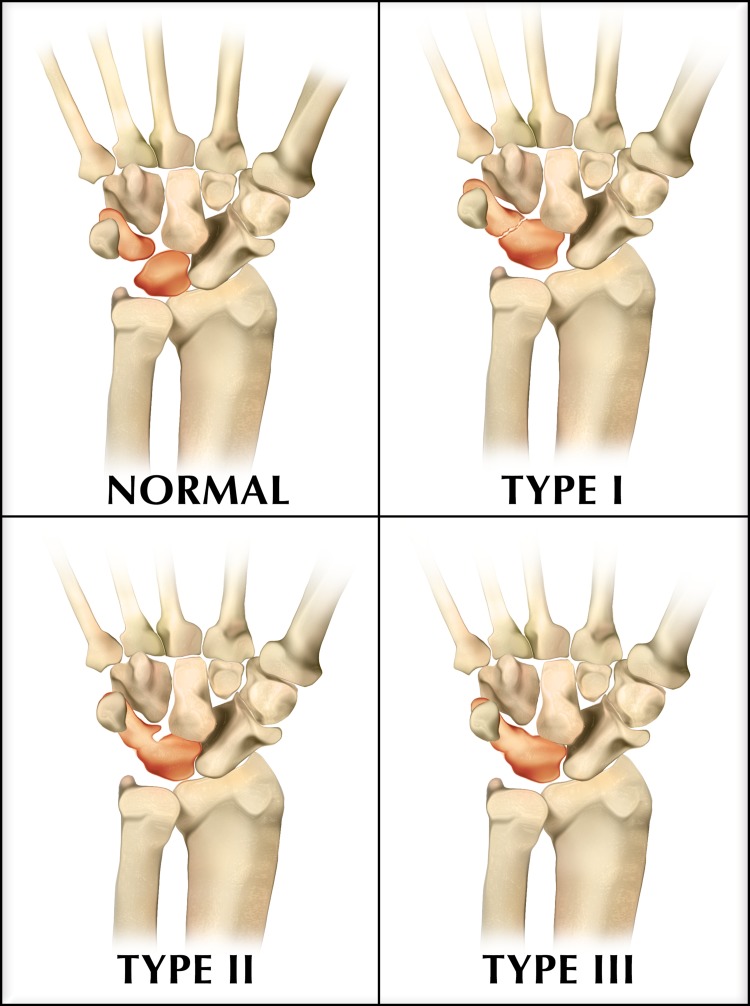
Illustration of Minnaar (or De Villiers Minnaar) classification system of lunotriquetral coalition Type I is an incomplete fibrocartilaginous coalition. Type II is an incomplete osseous coalition proximally with a distal cleft. Type III is a complete lunotriquetral osseous coalition. Type IV (not shown) is a complete lunotriquetral osseous coalition, as in type III, with other carpal abnormalities.

Carpal coalitions can be broadly classified as partial or complete and osseous or non-osseous or they can be classified more specifically by the type of tissue uniting the carpal bones: osseous coalition (with a synostosis between the two bones), fibrous coalition (with a syndesmosis) or cartilaginous coalition (with a synchondrosis) [1,3]. The Minnaar (or De Villiers Minnaar) classification system divides lunotriquetral coalitions into four types (Figure 4). Type I is an incomplete fibrocartilaginous coalition. Type II is an incomplete osseous union proximally with a distal cleft. Type III is a complete lunotriquetral osseous coalition, and type IV is a complete lunotriquetral osseous coalition with other carpal bone abnormalities [6]. Partial (type II) and complete (type III) osseous coalitions are the most common types, representing approximately 22% and 75% of lunotriquetral coalitions, respectively [6]. Types I and IV are much less common, representing only approximately 2% and 1% of lunotriquetral coalitions, respectively [6].

Complete osseous union at a carpal coalition is considered by most to be asymptomatic and is found incidentally at imaging for other causes of pain or after a trauma [3]. However, there are several case reports and small case series in the orthopedic and adult radiology literature which detail symptomatic non-osseous partial lunotriquetral coalitions (Minnaar type I coalitions) causing ulnar-sided wrist pain [2-7]. The exact reason why Minnaar type I partial coalitions lead to symptoms is unknown. However, it is speculated that the portion of the formed joint is covered by inadequate articular cartilage, causing it to be prone to early arthritis [6]. This is thought to be the reason why some of these patients do not tolerate stress loading or trauma at the lunotriquetral coalition, either of which can lead to the bone marrow edema-like signal and subchondral cystic changes in the lunate and triquetrum adjacent to the coalition that may be seen at imaging [3].

We found only two reports of children with symptomatic lunotriquetral coalitions in the English medical literature, a 15-year-old girl and a 14-year-old girl [2,6]. To the best of our knowledge, this is the first reported case of a symptomatic lunotriquetral coalition in a boy. The radiographs shown in the reports for both girls revealed obvious fibrocartilaginous coalitions similar to the radiographic findings seen in the adult patients with symptomatic lunotriquetral coalitions [2-7]. These girls were more skeletally mature than the boy we present. This is expected given that our patient was only 13 years of age when his radiographs were performed and that girls typically are more skeletally mature than boys of the same age. This is likely the reason why his coalition was not detected on that exam and is in accordance with the findings of a recent paper describing the evolution of the radiographic appearance of carpal coalitions [8]. The carpal bones begin as unossified cartilage and then progressively ossify during childhood; therefore carpal coalitions will be radiographically occult in early childhood. Pruszczynski et al. evaluated serial radiographs in children with carpal coalitions and found lunotriquetral coalitions first become apparent on radiographs between seven and thirteen years of age [8]. The earliest radiographic findings are close apposition of the lunate and triquetrum with a "parallel cortex" appearance [8]. They also found that this transition between radiographically undetectable to detectable lunotriquetral coalitions may occur rapidly and reported a case of a 13-year-old who had sequential radiographs which showed this occurring over a two-month period [8].

**Figure 5:**
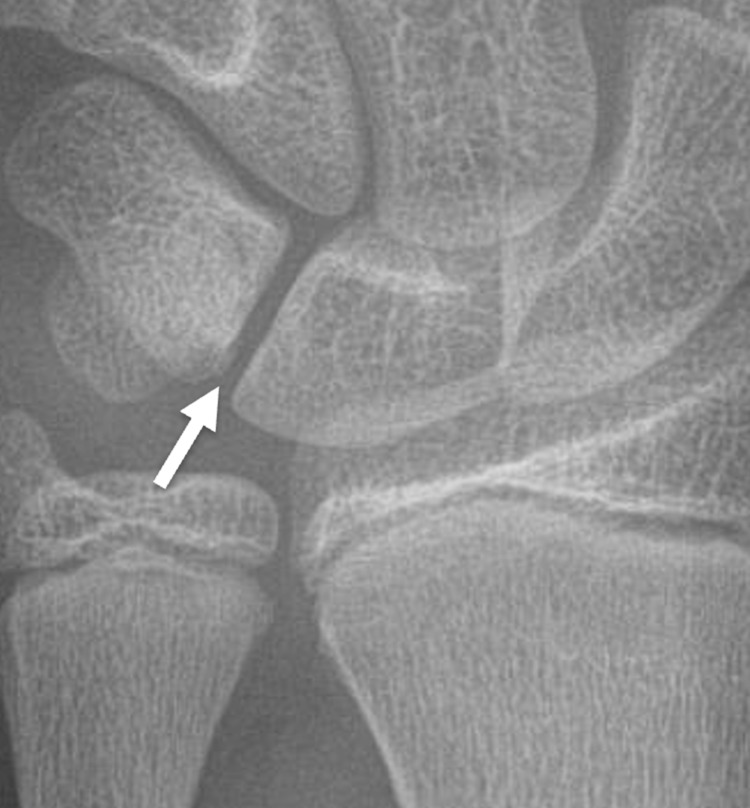
Magnified image of the same child's initial frontal wrist radiograph There are very early radiographic findings of a lunotriquetral coalition with close apposition of the proximal aspects of the lunate and triquetrum with a ‘parallel cortex’ appearance (arrow).

The radiographs of the 13-year-old boy that we present were initially interpreted as normal. In retrospect, close inspection of a magnified image from the frontal radiograph of that exam (as seen in Figure 5) shows the close apposition and the "parallel cortex" appearance that Pruszczynski et al. describe as the initial radiographic findings of a lunotriquetral coalition [8]. However, on an MRI performed two months after the radiographs the coalition is readily apparent. That MRI shows subchondral cystic changes and edema-like signal in the lunate and triquetrum adjacent to the coalition, which indicate stress changes. Other studies have reported similar MR findings of stress changes in patients with symptomatic partial fibrocartilaginous lunotriquetral coalitions [3,6]. To our knowledge, this is the first report of a symptomatic lunotriquetral coalition which did not have obvious radiographic findings at clinical presentation. This illustrates the utility of MRI in the diagnosis of symptomatic Minnaar type I coalitions, particularly in young patients whose radiographs may not suggest the diagnosis.

It is important to diagnose symptomatic Minnaar type I lunotriquetral coalitions because surgical treatment is typically indicated in patients whose symptoms do not resolve with conservative management. Partial wrist arthrodesis at the lunotriquetral joint has been shown to lead to resolution of symptoms and improved range of motion in adult patients [2,5-7]. Arthrodesis was also performed in the two children previously reported with symptomatic Minnaar type I coalitions with improvement of their symptoms [2,6]. 

## Conclusions

The majority of lunotriquetral coalitions are asymptomatic and are often first discovered by radiologists incidentally when imaging the wrist, hand or forearm for other reasons. Physicians caring for children need to be aware that a rare subset of lunotriquetral coalitions, partial fibrocartilaginous (Minnaar type I) coalitions, can be the cause of chronic ulnar-sided wrist pain. Furthermore, the case presented here illustrates that these may be radiographically occult or show only extremely subtle findings on radiographs in skeletally immature children. However, MRI can accurately demonstrate this type of coalition and show any associated stress changes and is therefore useful for diagnosis and preoperative planning.
